# Association between systolic blood pressure variability and severity of cerebral amyloid angiopathy in incident intracerebral hemorrhage

**DOI:** 10.3389/fstro.2023.1278610

**Published:** 2023-09-28

**Authors:** Tom J. Moullaali, Rachel Walters, Mark Rodrigues, Neshika Samarasekera, Jose Bernal, Xia Wang, Catherine Humphreys, Joanna M. Wardlaw, Andrew Farrall, Colin Smith, Craig S. Anderson, Rustam Al-Shahi Salman, Brian McKinstry

**Affiliations:** ^1^Centre for Clinical Brain Sciences, University of Edinburgh, Edinburgh, United Kingdom; ^2^Faculty of Medicine, The George Institute for Global Health, University of New South Wales, Sydney, NSW, Australia; ^3^German Centre for Neurodegenerative Diseases (DZNE), Magdeburg, Germany; ^4^Department of Neuropathology, Edinburgh Royal Infirmary, Edinburgh, United Kingdom; ^5^Global Brain Health, The George Institute China, Beijing, China; ^6^Usher Institute of Population Health Sciences and Informatics, University of Edinburgh, Edinburgh, United Kingdom

**Keywords:** intracerebral hemorrhage, blood pressure variability, cerebral amyloid angiopathy, autopsy (research), cohort study

## Abstract

**Introduction:**

The role of systolic blood pressure (SBP) variability in the pathogenesis of cerebral amyloid angiopathy (CAA) as an underlying cause of intracerebral hemorrhage (ICH) is unknown. We studied SBP variability before ICH according to CAA severity at autopsy.

**Methods:**

We collected office (primary care or hospital clinic) BP readings during 10 years before first-ever ICH onset in adults who died and had brain research autopsy in the Lothian IntraCerebral Hemorrhage, Pathology, Imaging, and Neurological Outcome (LINCHPIN), prospective, population-based, inception cohort study. A neuropathologist assessed CAA severity using a histopathological rating scale, masked to BP readings. Functional principal component analysis was used to model SBP levels by time before ICH, and logistic regression models assessed associations of SBP variability indices with CAA severity (moderate-severe vs. absent-mild) adjusted for age, gender, and mean SBP.

**Results:**

Among 72 adults (median age 81 [interquartile range 76–86], 56% female, median number of SBP readings 11 [3–19]), patients with moderate-severe CAA had similar mean SBP (143 vs. 145 mmHg, *P* = 0.588) but lower SBP variability (SBP standard deviation [SD] 14 vs. 17 mmHg, *P* = 0.033) compared with patients with absent-mild CAA, and their SBP trajectories seemed to differ over 10 years before ICH. The odds of moderate-severe CAA were higher with lower maximum SBP (adjusted OR per 10 mmHg lower: 1.53, 95% confidence interval [CI] 1.09–2.15; *P* = 0.015) and lower SBP range (1.29 [1.03–1.61]; *P* = 0.028), but not SBP SD (1.95 [0.87–4.38]; *P* = 0.11).

**Discussion:**

Compared with absent-mild autopsy-verified CAA, moderate-severe CAA is associated with lower maximum and range of pre-morbid SBP.

## Introduction

Two cerebral small vessel disease (CSVD) subtypes are among the commonest causes of spontaneous intracerebral hemorrhage (ICH; Al-Shahi Salman et al., 2009): arteriolosclerosis (also known as age-related deep-perforating arteriopathy), which can occur anywhere in the brain, and cerebral amyloid angiopathy (CAA), which is confined to cortical and leptomeningeal vessels (Samarasekera et al., 2012).

High blood pressure (BP) is an important modifiable risk factor for CSVD-associated ICH (O'Donnell et al., 2016). The Perindopril Protection Against Recurrent Stroke Study (PROGRESS) showed that in patients with a history of stroke (including ICH), treatment with an angiotensin-converting-enzyme inhibitor with or without a diuretic lowered average systolic blood pressure (SBP) levels and reduced the risk of future CSVD-associated ICH by 50% compared with placebo (Weinberger, 2001). Notably, there was no apparent heterogeneity in the treatment effect according to the predicted underlying CSVD subtype underlying the ICH during follow-up (non-CAA vs. CAA using MRI criteria; Knudsen et al., 2001; Arima et al., 2010).

A population-based study of SBP levels before incident stroke reported differences in premorbid SBP levels according to ICH location, with lower mean and maximum SBP levels observed in people with lobar ICH location compared to people with deep or infratentorial ICH location (Fischer et al., 2014). Higher and more variable SBP (SBPV variability, SBPV) being associated with incident cardiovascular disease, stroke, and renal impairment (Stevens et al., 2016), and the severity and progression of CVSD rated on brain imaging (Ma et al., 2020; Tully et al., 2020). However, direct evidence about the role of high or variable SBP (SBP variability, SBPV) in the pathogenesis of CAA is limited (Banerjee et al., 2017).

We hypothesized that differences in SBP levels before ICH might exist according to underlying CVSD subtype, with CAA-related ICH being associated with lower and less variable SBP compared with non-CAA related ICH (i.e., arteriolosclerosis-related ICH, driven by standard vascular risk factors including high and variable SBP). Therefore, we aimed to determine the association between long-term visit-to-visit SBPV in the preceding 10 years according to the severity of CAA verified at autopsy after ICH.

## Materials and methods

### Setting: lothian intracerebral hemorrhage, pathology, imaging, and neurological outcome study

The Lothian Audit of the Treatment of Cerebral Hemorrhage (LATCH) was a prospective population-based registry that identified all adults (age ≥16 years) with incident symptomatic stroke due to ICH whilst resident in the Lothian region of Scotland, UK, between 1 June 2010 and 31 May 2013, inclusive (Samarasekera et al., 2015a). The Lothian IntraCerebral Hemorrhage, Pathology, Imaging, and Neurological Outcome (LINCHPIN) study was a nested prospective community-based research study examining the causes of ICH using research autopsy in the case of death. The design and methods of the LINCHPIN study are described in detail elsewhere (Samarasekera et al., 2015b).

In brief, patients with incident ICH ascertained by LATCH were invited to consent to research autopsy where a neuropathologist (CS) took 1 cm cubed brain tissue samples from all lobes of the brain, the cerebellum and brainstem for research purposes; the remainder of the brain was returned to the donor's body. For patients who lacked mental capacity, consent was sought from their nearest relative or a legal representative in accordance with the statutory requirements of the Human Tissue (Scotland) Act 2006. The Scotland A Research Ethics Committee approved the study (10/MRE00/23).

### Participants

Patients with incident first-ever CSVD-associated ICH; a symptomatic event (new headache, neurological symptoms, or altered consciousness), referable to a discrete parenchymal bleed (confirmed by temporally consistent radiology or pathology findings). For the present analysis, patients were included if they participated in the LINCHPIN study and had research autopsy. We excluded cases of recurrent ICH, isolated extra-axial intracranial hemorrhage, and ICH definitely attributable to a macrovascular cause (e.g., arteriovenous or cavernous malformation, cerebral aneurysm, venous thrombosis), tumor, trauma, and hemorrhagic transformation of ischemic stroke.

We extracted baseline demographic and clinical characteristics of patients with incident first-ever ICH from the LATCH database, including age and sex, cardiovascular disease diagnoses and risk factors, medications prescribed at the time of ICH diagnosis (BP-lowering, antiplatelet and anticoagulant medications), and baseline BP and Glasgow coma scale (GCS) score.

### BP data collection

We collected up to six office (primary care or hospital clinic) BP readings per year during 10 years before the time of onset of ICH from paper and electronic healthcare records, and entered these data into the LATCH database according to standard operating procedures.

### Patient-level long-term visit-to-visit SBP levels and SBPV

We calculated the following summary measures of patient-level long-term visit-to-visit SBP levels and SBPV during 10 years before ICH onset for patients with available BP data: mean and standard deviation (SD, primary summary measure of SBPV); secondary measures of SBPV, including: maximum; minimum; range; coefficient of variation (CV) = SD/mean × 100; and residual standard deviation (RSD) = SD adjusted for change in SBP over time.

### Pathological characteristics on research autopsy

We used pathological characteristics about patients enrolled in LINCHPIN who died during study follow-up and consented to autopsy examination by a neuropathologist (CS). A consensus histopathological rating scale was used for CAA, which was rated absent, mild, moderate-severe (Supplementary material).

### Statistical analysis

We conducted analyses according to a pre-specified protocol. We present summary data by frequency (%) for categorical variables, and mean (SD) or median (interquartile range, IQR) for continuous variables. To identify potential confounders of the association between lower long-term visit-to-visit SBPV with severity of underlying CAA, we compared baseline demographic and clinical characteristics of the cohort after stratification by absent-mild vs. moderate-severe CAA on research autopsy. Next, we compared the summary measures of patient-level long-term visit-to-visit SBPV during 10 years before ICH by absent-mild vs. moderate-severe vs. CAA on research autopsy. We present all univariable comparisons using the Chi squared test for categorical data, and the Kruskal–Wallis rank test for continuous data. Finally, we assessed associations of summary measures of lower long-term visit-to-visit SBPV during 10 years before ICH per 10 mm Hg *decrease* (continuous variable) in logistic regression models adjusted for mean SBP and potential confounders identified in univariable analyses.

We used STATA version 16.1 (Stata Press, 2019) in all analyses. We used FPCA in MATLAB R2019a (The Mathworks Inc., 2019) to identify the average (mean) pattern of long-term visit-to-visit SBP levels during 10 years before ICH onset, stratified by absent-mild vs. moderate-severe vs. CAA on research autopsy. Functional principal component analysis (FPCA) can be applied to sparse longitudinal SBP data to provide a graphical representation of average patterns of SBP over time for illustrative purposes (Shen et al., 2017).

## Results

There were 72 patients who participated in the LINCHPIN study and had research autopsy data available by 16 December 2019 (median age 81 [IQR 76–86], 56% female, median number of BP readings 11 [3–19]). Of these, 69 (96%) had BP data available during 10 years before ICH onset; 62 (86%) had ≥2 BP readings required for calculation of the primary summary measure of long-term visit-to-visit SBPV (SD of SBP).

Table 1 shows the characteristics of patients with incident first-ever ICH, stratified by severity of CAA on research autopsy (absent-mild vs. moderate-severe). Compared with patients with absent-mild CAA, patients with moderate-severe CAA were more likely to have lobar ICH location and less likely to have deep or infratentorial ICH location (85 vs. 32% and 15 vs. 68%, *p* < 0.001, respectively), and have lower systolic and diastolic BP at presentation (163 vs. 179 mm Hg, *p* = 0.023 and; 84 vs. 96 mm Hg, *p* = 0.027, respectively). There were no other significant differences in baseline demographic and clinical characteristics. Nor was there a statistically significant difference between median time from ICH to research autopsy. The numerical differences in time from ICH to autopsy (63 vs. 15 days for moderate-severe CAA vs. absent-mild CAA, respectively, *p* = 0.31) probably reflect the poorer prognosis from deep ICH amounting to earlier death and earlier autopsy on average, compared with lobar CAA-related ICH. These differences did not affect technical factors related to assessment of CAA severity at autopsy.

**Table 1:** Characteristics of patients with incident first-ever intracerebral hemorrhage, stratified by severity of cerebral amyloid angiopathy on research autopsy (LINCHPIN autopsy study, *N* = 72).

	**Cerebral amyloid angiopathy rating**		***P* ^∧^**
	**Absent-mild (*****N*** = **38)**	**Moderate-severe (*****N*** = **34)**	
Median time from ICH to autopsy, days	15 (7–519**)**	63 (9–628)	0.30
**Demographics**			
Age at onset, years	82 (75–87)	81 (76–85)	0.76
Sex, female	20 (53)	20 (59)	0.60
**Clinical characteristics at diagnosis**			
**Medical history**			
Hypertension	27 (71)	18 (53)	0.11
Atrial fibrillation	13 (34)	6 (18)	0.11
Myocardial infarction	2 (5)	3 (9)	0.55
Ischemic stroke	5 (13)	5 (14)	0.85
Transient ischemic attack	5 (13)	3 (9)	0.56
Diabetes mellitus	5 (13)	2 (6)	0.30
Peripheral vascular disease	3 (8)	0 (0)	0.10
Hyperlipidemia	5 (13)	6 (18)	0.60
Dementia	5 (13)	9 (26)	0.15
Smoking, current or ex	18 (47)	17 (50)	0.82
Alcohol intake, units per week	0 (0–13)	2 (0–7)	0.97
**Medications**			
BP-lowering			
None	17 (45)	20 (59)	0.42
One	10 (26)	8 (24)	
Many	11 (29)	6 (18)	
Antiplatelet			
None	16 (42)	19 (56)	0.11
One	22 (58)	13 (38)	
Many	0 (0)	2 (6)	
Anticoagulant			
None	32 (84)	30 (88)	0.62
One	6 (16)	4 (12)	
**Clinical assessment**			
Systolic BP, mm Hg	179 (32)	163 (23)	0.023
Diastolic BP, mm Hg	96 (23)	84 (15)	0.027
GCS score	12 (9–14)	13 (11–14)	0.45
**ICH location** ^*^			
Lobar	12 (32)	28 (85)	< 0.001
Deep or infratentorial	26 (68)	5 (15)	

Data are number (%), mean (standard deviation), or median (interquartile range).

∧ Chi-squared test for categorical data; Kruskal–Wallis test for continuous data.

* One participant with moderate-severe cerebral amyloid angiopathy on research autopsy did not have diagnostic imaging available.

**Figure 1:**
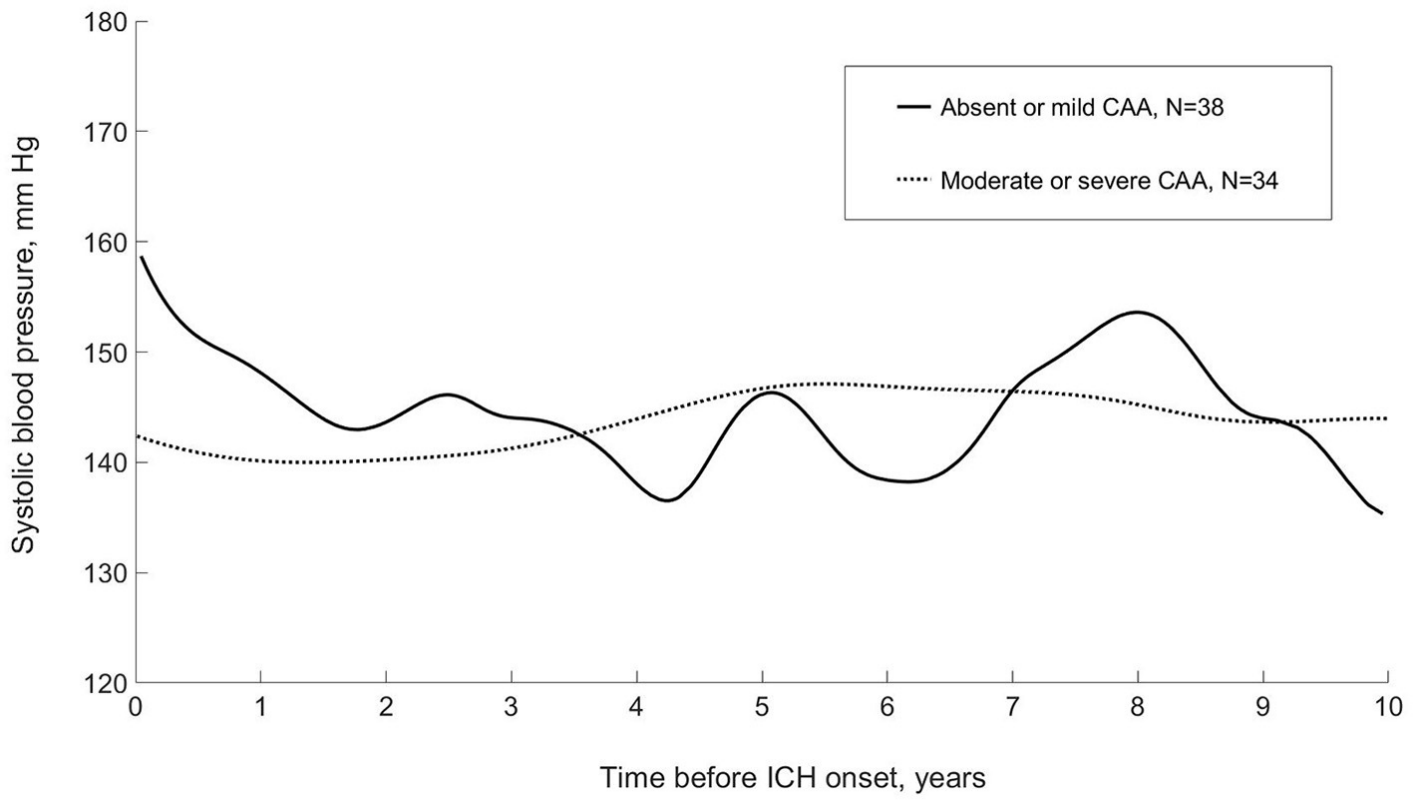
Systolic blood pressure during 10 years before incident first-ever intracerebral hemorrhage, stratified by severity of cerebral amyloid angiopathy on research autopsy: modeled using functional principle component analysis (LINCHPIN, *N* = 72). CAA, cerebral amyloid angiopathy; ICH, intracerebral hemorrhage.

When SBP data were modeled using a functional data analysis method and stratified by absent-mild vs. moderate-severe CAA on research autopsy, there appeared to be qualitative differences in the patterns of long-term visit-to-visit SBP during 10 years before ICH onset (Figure 1). Compared with patients with absent-mild CAA, patients with moderate-severe CAA had similar mean SBP (143 [13] mmHg vs. 145 [SD 15] mmHg from a median of 8 vs. 11 BP readings; *p* = 0.59 and *p* = 0.25, respectively) but lower patient-level summary measures of long-term visit-to-visit SBPV (Table 2).

**Table 2:** Patient-level long-term visit-to-visit systolic blood pressure variability during 10 years before incident first-ever intracerebral hemorrhage, stratified by severity of cerebral amyloid angiopathy on research autopsy (LINCHPIN autopsy study, *N* = 72).

**Patient-level variable**	**Cerebral amyloid angiopathy rating** ^*^		***P* ^∧^**
	**Absent-mild (*****N*** = **38)**	**Moderate-severe (*****N*** = **34)**	
Total BP readings, median (IQR)	11 (5–23)	8 (2–16)	0.25
Two or more BP readings^$^	33 (87)	29 (85)	0.85
Three or more BP readings^#^	30 (79)	25 (74)	0.59
**Systolic BP, mm Hg**			
Mean, mean (SD)	145 (15)	143 (13)	0.59
**SBPV summary measure, mean (SD)**			
SD (primary)	17 (6)	14 (8)	0.033
Minimum	121 (18)	125 (20)	0.51
Maximum	173 (25)	161 (20)	0.040
Range	57 (25)	42 (25)	0.017
CV	12 (4)	10 (5)	0.050
RSD	17 (6)	14 (7)	0.032

Data are number (%), mean (standard deviation), or median (interquartile range).

BP, blood pressure; SD, standard deviation; CV, coefficient of variation; RSD, residual standard deviation.

* From consensus histopathological rating for cerebral amyloid angiopathy.

∧ Chi-squared test for categorical data; Kruskal–Wallis test for continuous data.

$ Minimum number of readings required to calculate SD of systolic BP.

# Minimum number of readings required to calculate RSD of systolic BP.

In binary logistic regressions model adjusted for age, sex and mean SBP, every 10 mmHg decrease in SD of SBP during 10 years before ICH onset was associated with a non-significant 95% increase in the odds of moderate-severe CAA (adjusted OR [95%CI]: 1.95 [0.87–4.38], *p* = 0.11; Table 3 model 2). Of the secondary summary measures of long-term visit-to-visit SBPV during 10 years before ICH onset, only lower maximum and range of SBP were significantly associated with moderate-severe CAA after adjustment for mean SBP (adjusted OR per 10 mm Hg decrease in the summary measure: 1.53 [1.09–2.15], *p* = 0.015 and; 1.29 [1.03–1.61], *p* = 0.028, respectively, Table 3 model 2).

**Table 3:** Association between patient-level long-term visit-to-visit systolic blood pressure variability during 10 years before incident first-ever intracerebral hemorrhage and moderate-severe cerebral amyloid angiopathy on research autopsy (LINCHPIN autopsy study, *N* = 72).

**Systolic BP, mm Hg**	**Model 1**			**Model 2**		
	aOR^*^	**95% CI**	***P***	aOR^*^	**95% CI**	***P***
Mean	1.13	0.79–1.61	0.49	–	–	–
**SBPV summary measure**						
SD (primary)	2.05	0.92–4.54	0.079	1.95	0.87–4.38	0.11
Minimum	0.89	0.69–1.15	0.44	0.71	0.49–1.02	0.066
Maximum	1.30	1.02–1.64	0.029	1.53	1.09–2.15	0.015
Range	1.29	1.03–1.61	0.023	1.29	1.03–1.61	0.028
CV	2.58	0.79–8.39	0.12	2.61	0.79–8.59	0.12
RSD	2.30	0.90–5.87	0.080	2.14	0.78–5.85	0.14

BP, blood pressure; CI, confidence interval; CV, coefficient of variation; aOR, adjusted odds ratio; RSD, residual standard deviation; SD, standard deviation.

^*^Per 10-point decrease in the systolic BP summary measure.

Model 1: binary logistic regression model adjusted for age and sex.

Model 2: binary logistic regression model adjusted for covariables in Model 1 and mean systolic BP.

## Discussion

In this population-based cohort which included 72 patients with ICH who had research autopsy, several summary measures indicating lower long-term visit-to-visit SBPV before ICH (lower maximum and smaller range, but not SD of SBP), were associated with moderate-severe CAA, independently of age, sex, and mean SBP.

High BP has long been recognized as an important risk factor for non-lobar ICH (Brott et al., 1986), where the underlying pathology is principally arteriolosclerosis (non-CAA CSVD; Rodrigues et al., 2018), However, accumulating evidence indicates that high BP is also a risk factor for lobar ICH (Arima et al., 2010; Biffi et al., 2015), whereby non-CAA and CAA CSVD exist independently or co-exist, to varying degrees (Rodrigues et al., 2018). Yet, differences in premorbid BP control seemed to exist according to ICH location in a previous population-based study, with lower mean and maximum premorbid SBP levels observed in patients with lobar compared to deep or infratentorial ICH (Fischer et al., 2014). Our findings indicate these differences may be explained by the principal CSVD subtype underlying incident ICH.

Current models of non-CAA and CAA pathogenesis may provide a basis for this. In the brain, higher intracranial pulsatility and reduced cerebral vasoreactivity are manifestations of increased arterial stiffness, and these variables are among several that may contribute to the pathogenesis of non-CAA CSVD (arteriolosclerosis; Banerjee et al., 2017; Wardlaw et al., 2019). In the systemic circulation, long-term visit-to-visit SBPV is associated with systemic arterial stiffness (Tedla et al., 2017), cardiovascular morbidity and mortality (Stevens et al., 2016), and the presence of CSVD on brain imaging (Tully et al., 2020). Thus, arterial stiffness may be a common mechanistic factor for the development of non-CAA CSVD and high long-term visit-to-visit SBPV. It is less clear, though, as to whether arterial stiffness contributes to the pathogenesis of CAA, where there are changes in cerebral hemodynamics that can be attributed to amyloid-β deposition in the cerebrovascular vessel wall, and not the other way around (Banerjee et al., 2017). The finding that ICH patients with underlying moderate-severe CAA appear to have lower maximum and range of SBP during 10 years before onset of the ictus supports there being a smaller contribution to the pathogenesis of CAA from factors indicating vascular stiffness. This is an early step to understanding the role of BP in the pathogenesis of CAA, where few data were previously available (Banerjee et al., 2017).

The strengths of this study include the prospective, population-based design and completeness of demographic, clinical, radiological, BP, and histopathological data according to standard definitions. However, reliance on histopathological data limited the sample size for analysis, reducing both power to detect associations and an ability to fully adjust for multiple confounders. Further, meaningful analyses of the association between higher long-term visit-to-visit SBP levels and SBPV before ICH and the severity of non-CAA CVSD were hampered by the absence of histopathological ratings that adequately discriminate non-CAA CSVD severity in this cohort. Therefore, any conclusions about potential differences in SBP levels and SBPV according to CSVD subtype are limited by the reliance on the assumption that ICH in the context of absent-mild underlying CAA was likely to be related to non-CAA CVSD.

Further data are required to test the robustness of these preliminary findings about long-term visit-to-visit SBPV before ICH. To detect a true difference in the role of long-term visit-to-visit SBPV in the pathogenesis of the two principal CSVD subtypes that cause ICH, larger patient and BP sample sizes are required. In the absence of large samples of histopathological data, external validation of the Edinburgh CT criteria for lobar ICH associated with CAA is a prerequisite for any future analyses that might aim to pool patient-level data from several rigorous population-based studies of ICH (Wolfe et al., 2002; Lovelock et al., 2007; Samarasekera et al., 2015a). Novel approaches to data-linkage may harness larger and more representative samples of BP data from various forms of BP tele-monitoring and wearable devices. The association of blood pressure control, including SBPV, with cognitive impairment associated with CAA also warrants further investigation.

Regarding non-CAA SVD, histopathological ratings of non-CAA CSVD that better represent the spectrum of this disease in ICH patients are needed to assess the association between long-term visit-to-visit SBPV before ICH and factors that indicate the presence of cerebral vessels at risk of rupture, such as lipohyalinosis and fibrinoid necrosis.

In conclusion, people with ICH who had moderate-severe CAA at autopsy had lower maximum and range of SBP before ICH compared to people with ICH with absent-mild CAA. These findings support there being differences in the role of BP control in the pathogenesis of the two main CSVD subtypes that cause ICH.

## Data availability statement

The raw data supporting the conclusions of this article will be made available by the authors, without undue reservation.

## Ethics statement

The studies involving humans were approved by Scotland A Research Ethics Committee. The studies were conducted in accordance with the local legislation and institutional requirements. The participants provided their written informed consent to participate in this study.

## Author contributions

TM: Conceptualization, Formal analysis, Funding acquisition, Investigation, Writing—original draft, Writing—review and editing. RW: Data curation, Writing—review and editing. MR: Data curation, Writing—review and editing. NS: Conceptualization, Data curation, Investigation, Writing—review and editing. JB: Formal analysis, Writing—review and editing. XW: Conceptualization, Formal analysis, Writing—review and editing. CH: Data curation, Writing—review and editing. JW: Data curation, Writing—review and editing. AF: Data curation, Writing—review and editing. CS: Data curation, Writing—review and editing. CA: Supervision, Writing—review and editing. RA-S: Conceptualization, Data curation, Funding acquisition, Methodology, Supervision, Writing—review and editing. BM: Conceptualization, Methodology, Supervision, Writing—review and editing.
