# Supplementary material for: Association between systolic blood pressure variability and severity of cerebral amyloid angiopathy in incident intracerebral hemorrhage
Source: Front Stroke. 2023 Sep 28;2:1278610. doi: 10.3389/fstro.2023.1278610 (PMC12802715; doi:10.3389/fstro.2023.1278610)
Supplement: Supplementary file 1 [file Data_Sheet_1.DOCX]

**ONLINE SUPPLEMENT**

**Consensus histopathological rating scale for cerebral small vessel disease**^1^

| Score | Parenchymal CAA | Meningeal CAA | Capillary CAA | Vasculopathy |
| --- | --- | --- | --- | --- |
| 0 | Absent | Absent | Absent | Absent |
| 1 | Scant β-amyloid deposition | Scant β-amyloid deposition | Present | Occasional vessel |
| 2 | Some circumferential β-amyloid | Some circumferential β-amyloid |  | Many vessels |
| 3 | Widespread circumferential β-amyloid | Widespread circumferential β-amyloid |  |  |

CAA = cerebral amyloid angiopathy.

Reference:

1. Love S, Chalmers K, Ince P, et al. Development, appraisal, validation and implementation of a consensus protocol for the assessment of cerebral amyloid angiopathy in post-mortem brain tissue. Am. J. Neurodegener. Dis. 2014;3(1):19–32.
